# Hippocampal expression of Wnt7a and β-catenin in depression: evidence from chronic unpredictable mild stress

**DOI:** 10.7717/peerj.20837

**Published:** 2026-02-19

**Authors:** Zehao Zhang, Jialong Huang, Hongyue Yu, Zi jun Ji, Zijuan Ding, Yiting Wang, Jinyu Kang, Zhen Li, Zhuxin Sui

**Affiliations:** 1 College of Clinical Medicine, Qilu Medical University, Zibo, Shandong, China; 2 College of Medical Imaging, Qilu Medical University, Zibo, Shandong, China; 3 College of Dentistry, Qilu Medical University, Zibo, Shandong, China; 4 Undergraduate Academic Affairs Office, Qilu Medical University, Zibo, Shandong, China; 5 School of Basic Medical Sciences, Qilu Medical University, Zibo, Shandong, China

**Keywords:** Chronic unpredictable mild stimulation, Hippocampus, Depression, Wnt7a/β-catenin signaling cascade, Wnt7a

## Abstract

This study sought to examine the impact of Wnt7a/β-catenin signaling on depressive-like behaviors by using a rodent model subjected to chronic unpredictable mild stress (CUMS). Hippocampal Wnt7a and β-catenin expression levels were analyzed to investigate their mechanistic involvement in depression. Therefore, 20 male Sprague-Dawley rats were randomly allocated to the control or the CUMS experimental groups. The CUMS group underwent a 30-day stress protocol involving randomized stimuli. This study was authorized by the Ethics Committee (approval no. YXLL2022006). Following model establishment, depression-related behavioral phenotypes were quantitatively evaluated using standardized behavioral paradigms, the sucrose preference test (SPT), and the open field test (OFT), targeting core symptom domains such as anhedonia and alterations in locomotor activity. The morphology of hippocampal CA2 and DG area neurons was examined using hematoxylin and eosin staining, while immunofluorescence and Western blotting assessed Wnt7a and β-catenin expression. Western blotting also assessed GSK-3β and p-GSK-3β expression. Results indicated that CUMS rats showed markedly lower SPT indices (*P* < 0.05) and decreased OFT parameters (total distance traveled, central zone activity, speed, and central zone duration) *versus* controls (*P* < 0.05). Notably, Wnt7a, β-catenin, GSK-3β, and p-GSK-3β were significantly upregulated in the hippocampal tissues of rats in CUMS group (*P* < 0.05). Collectively, this study found that CUMS-induced depression is associated with a significant upregulation of hippocampal Wnt7a, β-catenin, and GSK-3β, along with increased GSK-3β phosphorylation. This correlative evidence points to Wnt pathway activation in depression pathogenesis and warrants further mechanistic investigation.

## Introduction

Depression constitutes a heterogeneous condition marked by diverse emotional, cognitive, and behavioral manifestations. It is predominantly marked by a high prevalence, low treatment uptake, and recurrent episodes. By 2030, depression is projected to dominate as a primary global disease burden, intensifying pressures on healthcare systems worldwide (Zhao et al., 2024; Guo et al., 2025). The pathogenesis of depressive disorders involves complex biological mechanisms, with several theoretical frameworks proposed to explain its etiology. Most notably, the monoamine hypothesis encompasses the dysregulation of serotonin, noradrenaline, and dopamine systems in the pathogenesis of depression. Current research focuses on elucidating the mechanistic associations between dysregulated neural signaling pathways and the etiopathogenesis of affective disorders, particularly through molecular neuroscience approaches (Zhu et al., 2024; Yin et al., 2025; Guo et al., 2024). Accumulating experimental evidence has suggested that the Wnt/β-catenin signaling cascade is critically involved in the onset of depressive disorder, primarily through its regulatory effects on synaptic plasticity and neurogenesis (Xiao et al., 2024; Yin et al., 2024). The significance of this pathway in mental disorders is well recognized (Bem et al., 2019), with recent work further highlighting the role of Wnt-regulated adult hippocampal neurogenesis in affective functions (Alonso, Petit & Lledo, 2024). However, the current evidence on particular Wnt subtypes, which could be involved in the pathogenesis of depressive disorder, remains limited. Experimental findings from preclinical models suggest that Wnt1, Wnt2, and Wnt3 can mediate β-catenin-dependent signaling cascades to orchestrate neuroregulatory processes associated with the pathophysiology of affective disorders (Zhang et al., 2019; Zhou et al., 2016). Additionally, previous studies supported the involvement of Wnt signaling in neurodegenerative diseases, primarily through its regulatory role in amyloid-β aggregation and α-synuclein proteostasis (Purro, Galli & Salinas, 2014). Although the effect of Wnt7a-mediated β-catenin signaling in depressive pathophysiology has not been fully delineated, this evolutionarily conserved signaling axis is recognized as a key modulator of neuroplasticity and synaptic homeostasis (Zhang & Wang, 2020). Other studies have also revealed that Wnt7a can exert a multifaceted influence on the process of hippocampal neurogenesis in adults, primarily through regulating the developmental trajectory of newly generated neurons (Anand et al., 2023; Aghaizu et al., 2023). Extending these discoveries, we hypothesized that dysregulation in the β-catenin-dependent pathway may constitute a molecular mechanism underlying the pathogenesis of depression.

The current study employed chronic unpredictable mild stress (CUMS) to generate depressive-like phenotypes in rodents. Behavioral assessments included sucrose preference test (SPT) to evaluate anhedonia and open field test (OFT). The OFT was included to quantify anxiety-like behavior as a component of the depressive phenotype and as an essential control to rule out generalized locomotor deficits as a confounding factor for the observed reduction in sucrose consumption. Subsequently, conducting neurophysiological analysis using light microscopy and complementary methodologies to identify cytoarchitectural alterations in hippocampal neuronal networks. This investigation focused on the canonical Wnt signaling pathway, *via* employing molecular profiling to characterize the regulatory dynamics of core components, Wnt7a, and β-catenin. Exploratory analyses further explored the potential association between hippocampal morphometric alterations and dysregulation of the β-catenin-dependent signaling axis. Overall, this study aimed to assess the possible functional association between hippocampal structural changes and β-catenin-mediated signaling activity, thus establishing an empirical foundation for elucidating molecular mechanisms underlying the pathogenesis of depression.

## Materials & Methods

### Animal experiments

This study was conducted and reported in accordance with the ARRIVE 2.0 guidelines. 20 male SPF Sprague-Dawley rats weighing 180–200 g were sourced from Jinan Ponyue Co., Ltd. (nationally accredited facility, animal use certification no. SYXK(LU) 20230002). Multiple interventions were implemented throughout the study to minimize pain, suffering, and distress in accordance with the 3R principle of Refinement. All procedures were approved by the Medical Ethics Committee of Qilu Medical University (Approval No. YXLL2022006). Upon arrival, rats were group-housed (four per cage) under standard conditions at 21–25 °C with a 12/12-h light/dark cycle and ad libitum access to food and water; environmental enrichment consisting of wooden blocks and nesting material was provided in each cage. After a 7-day adaptation period, experimental procedures were initiated. Animals were monitored twice daily. Predefined humane endpoints requiring immediate euthanasia included: (1) >20% body weight loss within 48 h (baseline: 195 ± 5 g); (2) sustained anorexia (<5 g food/24 h for 48 h); (3) inability to right themselves within 30 s of gentle prodding; and (4) hemorrhage scoring ≥3 on a standardized scale (0 = no bleeding; 3 = persistent bleeding with tissue exposure). No animals met these criteria during the study. Before terminal procedures (perfusion and tissue collection), deep anesthesia was induced by intraperitoneal injection of 20% urethane (6 mL/kg, 1,200 mg/kg). The surgical plane of anesthesia was confirmed by the absence of pedal withdrawal reflex (upon applying atraumatic forceps pressure to the interdigital web for 2 s) and loss of corneal reflex (assessed by gentle touch to the corneal surface with a saline-moistened cotton wisp). Euthanasia was then performed by transcardial perfusion, with death confirmed by cessation of heartbeat and respiration. No surviving animals remained at the study’s conclusion.

### Major reagents and drugs

Six primary antibodies were commercially purchased by Hangzhou HuaAn Biotechnology Co., Ltd., including a rabbit polyclonal IgG, a recombinant rabbit monoclonal antibody targeting Wnt7a (Cat# ER1918-71), a recombinant rabbit monoclonal antibody targeting β-catenin (Cat# ET1601-5), a recombinant rabbit monoclonal targeting GSK-3β (Cat# ET1607-71), a recombinant rabbit monoclonal targeting phospho-GSK3 beta (S9) (Cat# ET1607-60) and a mouse monoclonal antibody against β-actin (Cat# HA722023). Additionally, a rabbit polyclonal antibody against Wnt7a (Cat# 27177-1-AP) was obtained from Wuhan Sanying Biotechnology for immunofluorescence staining. In addition, two secondary antibodies, namely donkey anti-rabbit IgG (cat. no. sc-362261) and donkey anti-rabbit IgG (cat. no. sc-362281), were obtained from Santa Cruz Biotechnology, Inc. (Dallas, TX, USA). An HRP-conjugated goat anti-rabbit IgG (Cat# HA1001; HuaAn Biotechnology, Hangzhou, China) was also used for western blotting.

### Grouping and CUMS modelling

**Figure 1:**
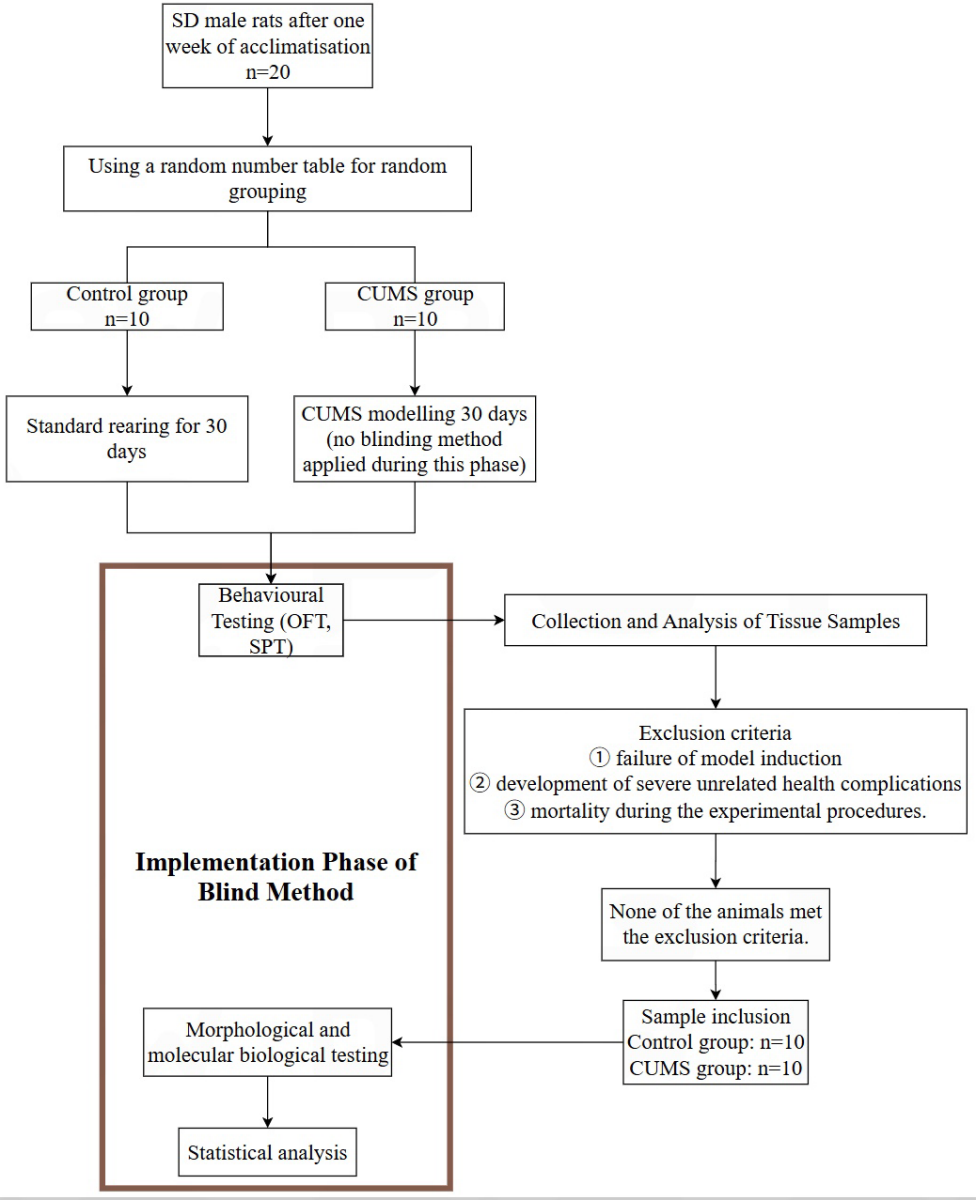
Schematic of the blinded evaluation process and animal exclusion.

The allocation of twenty rats to the control (*n* = 10) or CUMS (*n* = 10) groups was performed using a random number table by an investigator not involved in the subsequent experiments. This randomization strategy was complemented by randomizing cage locations and counterbalancing the order of all treatments and measurements daily to mitigate potential confounding effects. No anaesthesia was used on rats during the establishment of the CUMS model (*e.g.*, a “10-second plantar electrical stimulation”, which was administered as a single stimulus at a sub-threshold intensity (0.5 mA, 16 V) every 2 s to ensure it was non-noxious, eliciting aversion without pain, was administered without anesthesia) (Dai et al., 2010; Bai et al., 2020; Li et al., 2021). Inclusion criteria for animals were: male Sprague-Dawley rats weighing 180–200 g at the start of the experiment. Exclusion criteria, established *a priori*, were: (1) failure of model induction, (2) development of severe unrelated health complications, or (3) mortality during the experimental procedures. No animals, experimental units, or data points were excluded from any of the experimental groups during the analysis. All data from all 20 rats (10 per group) were included. Due to the nature of the CUMS procedure, which requires the application of specific stressors to the model group, the experimenter administering the daily stress protocols was necessarily aware of the group allocation. However, to minimize assessment bias, a different investigator, who was blinded to the group identity, performed all behavioral tests and data collection. The same blinding protocol was rigorously maintained during all molecular and histological analyses. Furthermore, the data analyst was also blinded during the statistical analysis (Fig. 1). Rats in the CUMS group underwent a 30-day established protocol including exposure to multi-modal stress (Table 1). This standardized procedure incorporated ten distinct stimuli. A single stressor was applied per day, delivered in a randomized and non-repetitive sequence according to a computer-generated schedule (Table 1), including 24-h cage tilting at 45° angle, 24-h exposure to damp bedding, 2-min tail pressure application, 24-h food/water deprivation, 5-min forced swimming in a 4 °C aqueous environment, 24-h exposure to ultrasonic frequency, continuous strobe light stimulation, 15-min horizontal cage agitation, and 10-sec plantar electrical stimulation. The entire set of ten stressors was repeated across three cycles throughout the 30-day period to maintain environmental unpredictability. Rats in the control group were maintained under standard housing conditions without experimental interventions. Following observation for 30 days, tissues were separately collected from both animal groups for subsequent experiments.

**Table 1:** CUMS modeling process. The schedule was generated by randomizing the sequence of ten distinct stressors, with the constraint that the same stressor would not be applied on consecutive days. One stressor was applied daily throughout the 30-day period.

Date	Stimuli	Date	Stimuli	Date	Stimuli
5.12	45° tilted cages	5.22	1d damp bedding exposure	6.1	10s plantar electrical stimulation
5.13	1d damp bedding exposure	5.23	forced swimming (4 °C, 5 min)	6.2	1d ultrasonic frequency exposure
5.14	2-min tail pressure application	5.24	15-min horizontal cage agitation	6.3	1d damp bedding exposure
5.15	1d water deprivation	5.25	24-hr ultrasonic frequency exposure	6.4	forced swimming (4 °C, 5 min)
5.16	10s plantar electrical stimulation	5.26	45° tilted cages	6.5	2-min tail pressure application
5.17	1d food deprivation	5.27	1d continuous photic stimulation	6.6	1d water deprivation
5.18	forced swimming (4 °C, 5 min)	5.28	1d food deprivation	6.7	15 min horizontal cage agitation
5.19	1d ultrasonic frequency exposure	5.29	2-min tail pressure application	6.8	45° tilted cages
5.20	1d continuous photic stimulation	5.30	1d water deprivation	6.9	1dr food deprivation
5.21	15 min horizontal cage agitation	5.31	10s plantar electrical stimulation	7.10	1d continuous photic stimulation

### Behavioral tests

The number of rats used for behavioral testing was *n* = 10 for the control group and *n* = 10 for the CUMS group. To assess anhedonia-like behaviors, following a 12-h fasting protocol with water restriction, rats were subjected to a sucrose preference test (SPT). Therefore, rats had simultaneous access to a 1% sucrose solution (10 g/l) and deionized water for 60 min. To eliminate spatial bias, the position of the bottles changed after 30 min. After the end of the experimental procedures, sucrose and fluid consumption were detected. The anhedonic index was mathematically calculated using the following formula: [sucrose consumption/ (sucrose + water consumption)] x 100%. Furthermore, the Open Field Test (OFT) was conducted to control for locomotor activity as a potential confound in the SPT and to concurrently assess anxiety-like behavior. OFT was conducted in the black-walled experimental arena measuring 120 × 120 × 35 cm, and allowed to explore freely for 5 min. All trials were performed under controlled acoustic conditions (background white noise at ∼60 dB) during consistent daily time intervals (*e.g.*, 17:00-22:00). Each rat was introduced into the central quadrant using tail-assisted positioning. Locomotion parameters, including total distance covered (a measure of general locomotor activity), time spent in the central zone that defined as the central 60 cm × 60 cm area of the arena (an indicator of anxiety-like behavior, with less time indicating higher anxiety), mean velocity, and central area movement patterns, were recorded using the Dr. Mouse behavioral tracking platform. The arena was thoroughly cleaned with a 75% ethanol solution and dried completely between trials to eliminate residual olfactory cues.

### Hematoxylin and eosin staining

Following anesthesia induction by intraperitoneal injection of 20% urethane 6 mL at a dosage of 6 mL/kg (1,200 mg/kg), four randomly selected rats from each group underwent thoracotomy to expose visceral organs. Subsequently, surgical access to the cardiac apex, hepatic vasculature, and aortic arch was established. A left ventricular puncture was performed into the ascending aorta, the right atrium was clipped, and then the liver underwent rapid perfusion with saline at room temperature until blanched, followed by 4 °C 4% paraformaldehyde perfusion (Zhang et al., 2025). The brain was then carefully extracted from the head, and it was fixed in 4% paraformaldehyde for 1d. Following fixation, the brain tissues were processed through a graded ethanol series, cleared in xylene, and embedded in paraffin blocks. Subsequently, coronal sections were cut at a thickness of 6 µm using a microtome, floated in a warm water bath to remove wrinkles, and mounted on glass slides, followed by drying overnight at 37 °C. For the staining procedure, the sections were first dewaxed in xylene and rehydrated through a graded ethanol series to distilled water. The sections were then stained with hematoxylin for 10 min, rinsed under running tap water for blueing, and briefly differentiated in 0.7% acid ethanol if necessary. After a further rinse, counterstaining was performed with eosin solution for 1–2 min. Finally, the sections were dehydrated through a graded alcohol series, cleared in xylene, and mounted with neutral balsam under coverslips.

To objectively quantify the observed reduction in neuronal density, morphologically intact neurons in the hippocampal CA2 and dentate gyrus (DG) subregions were counted. For each animal, a single, representative high-power field (HPF; 400x magnification) per region was analyzed. Neurons were classified as “normal” based on a clear, round-to-oval nucleus with a distinct nucleolus, intact nuclear membrane, and basophilic cytoplasm with defined contours. Neurons exhibiting pyknotic or fragmented nuclei, markedly reduced somatic size, and/or intensely eosinophilic cytoplasm were considered “abnormal” and excluded from the normal neuron count. Density is expressed as the number of normal neurons per HPF. This quantification was based on a representative single field rather than a full stereological assessment. The hippocampal structure was assessed under a microscope.

### Western blot analysis

For tissue processing, three rats per group were selected. Following anesthesia induction *via* intraperitoneal injection of 20% urethane, 6 mL at a dose of 6 mL/kg (1,200 mg/kg). Following decapitation, brains were rapidly excised, and hippocampi were dissected on ice. Total proteins were extracted by homogenizing the hippocampal tissues in an appropriate volume of RIPA lysis buffer (10:1, volume) supplemented with PMSF protease inhibitor, and the lysates were centrifuged (12,000× g, 15 min, 4 °C) to collect the supernatant. Protein concentration was quantified *via* a BCA assay. Subsequently, equal amounts of protein (20–30 µg) were separated by 10% SDS-PAGE and transferred onto PVDF membranes. For each sample, the separated proteins were transferred in parallel onto multiple PVDF membranes to allow for probing of different proteins on separate full membranes (one membrane for β-actin and others for target proteins). Immediately after transfer, the membranes were briefly rinsed with TBST to remove residual transfer buffer. Non-specific binding was obstructed by incubating membranes with 5% nonfat milk in TBS-Tween (TBST) for two hours at room temperature. The membranes were then washed three times with TBST for 10 min each. Membranes were probed overnight at 4 °C with primary antibodies against Wnt7a (rabbit; dilution, 1:500), β-catenin (rabbit; dilution, 1:1000), GSK-3β (rabbit; dilution, 1:1000), phospho-GSK-3β (rabbit; dilution, 1:5000), and β-actin (rabbit; dilution, 1:50000). Following 3*10 min washes in TBST thrice, the membranes were incubated with the corresponding HRP-conjugated goat anti-rabbit antibody (dilution, 1:5,000) for 1 h at indoor temperature, followed by another series of 3*10 min TBST washes. The protein bands were visualized using a low-background chemiluminescence substrate, and chemiluminescent signals were captured *via* a chemiluminescence imaging system. The band intensities for both target proteins and β-actin were quantified using ImageJ software. The normalized expression level of each target protein was calculated as the ratio of its band intensity to the β-actin band intensity from the same sample.

### Immunofluorescence staining

A total of six Sprague-Dawley male rats were randomly assigned to either the control or CUMS experimental groups (three rats per group). Animals were deeply narcotized *via* intraperitoneal injection of 20% urethane (6 ml/kg) at a dosage of 1,200 mg/kg, then transcardial perfusion with ice-cold 4% paraformaldehyde in 0.1 M PBS (pH = 7.4). Following decapitation, brains were carefully dissected and post-fixed in fresh 4% paraformaldehyde for 8–12 h at 4 °C, and were then cryoprotected by immersion in 30% sucrose-PBS solution at 4 °C for 72 h. Coronal brain sections (thickness, 20 µm) were prepared using a cryostat maintained at −20 °C. Furthermore, for immunofluorescence staining, free-floating sections were washed thrice (5 min each) with PBS containing 0.1% Triton X-100 (PBS-T). To block non-specific binding, the tissue sections were incubated for 2 h with blocking solution (5% bovine serum albumin in PBS-T) at indoor temperature. Then, the tissue sections were incubated with primary antibodies against WNT7a (rabbit polyclonal; dilution, 1:500) and β-catenin (rabbit monoclonal; dilution, 1:100), both diluted in blocking solution, at 4 °C overnight. Following three additional washes with PBS-T, sections were exposed to the corresponding fluorescent secondary antibodies (CF™488-conjugated donkey anti-rabbit IgG and CF™594-conjugated donkey anti-rabbit IgG; dilution, 1:200) for two hours at indoor temperature under light-protected conditions. Nuclei were counterstained with DAPI (dilution, 1:100) for 15 min and then mounted using antifade medium. Fluorescence imaging was performed using a laser-scanning confocal microscope with standardized acquisition parameters applied uniformly across all samples. The mean fluorescence intensity (MFI) of target proteins in the CA2 and DG subregions of the hippocampus was measured by ImageJ software.

### Statistical analysis

Data are presented as the mean ± standard deviation ($\bar {x}$ ± SD). All quantitative data, including behavioral test results and any other continuous outcome measures, were compared between the Control and CUMS groups using independent samples *t*-tests. For the analysis of Western blot data and Immunofluorescence data, which involved multiple related protein targets, we employed a two-way ANOVA to examine the effects of Treatment (Control *vs.* CUMS) and protein (the specific target) and their interaction. This was followed by Šídák’s multiple comparisons test to compare the two treatment groups within each protein. This approach controls the family-wise error rate associated with multiple comparisons. The normality of data distribution was assessed using the Shapiro–Wilk test, and the equality of variances was confirmed using Levene’s test.

For datasets that violated the assumption of normality, the non-parametric Mann–Whitney *U* test was employed instead. All statistical analyses were performed using SPSS software (version 19.0; SPSS, Inc., Chicago, IL, USA), GraphPad Prism software (version 9.5.0; GraphPad Software, Inc.), and G*Power software (version 3.1.9.7; G*Power software, Inc.). A two-tailed *P*-value of less than 0.05 was considered statistically significant. For the primary behavioral outcomes (*n* = 10 per group), statistical significance was assessed based on *p*-values, and the magnitude of the effects was quantified using Cohen’s d. Due to the constraints of a limited sample size in the secondary biochemical analyses (*n* = 3 per group, *e.g.*, Western blot and immunofluorescence), formal calculations of effect sizes with confidence intervals were not performed, as they would be highly unstable. The biological conclusions for these exploratory analyses are therefore supported primarily by the consistency of the observed effects across multiple experimental modalities, such as behavioral, histological, and biochemical.

## Results

### Behavioral assessment after CUMS modelling

**Figure 2:**
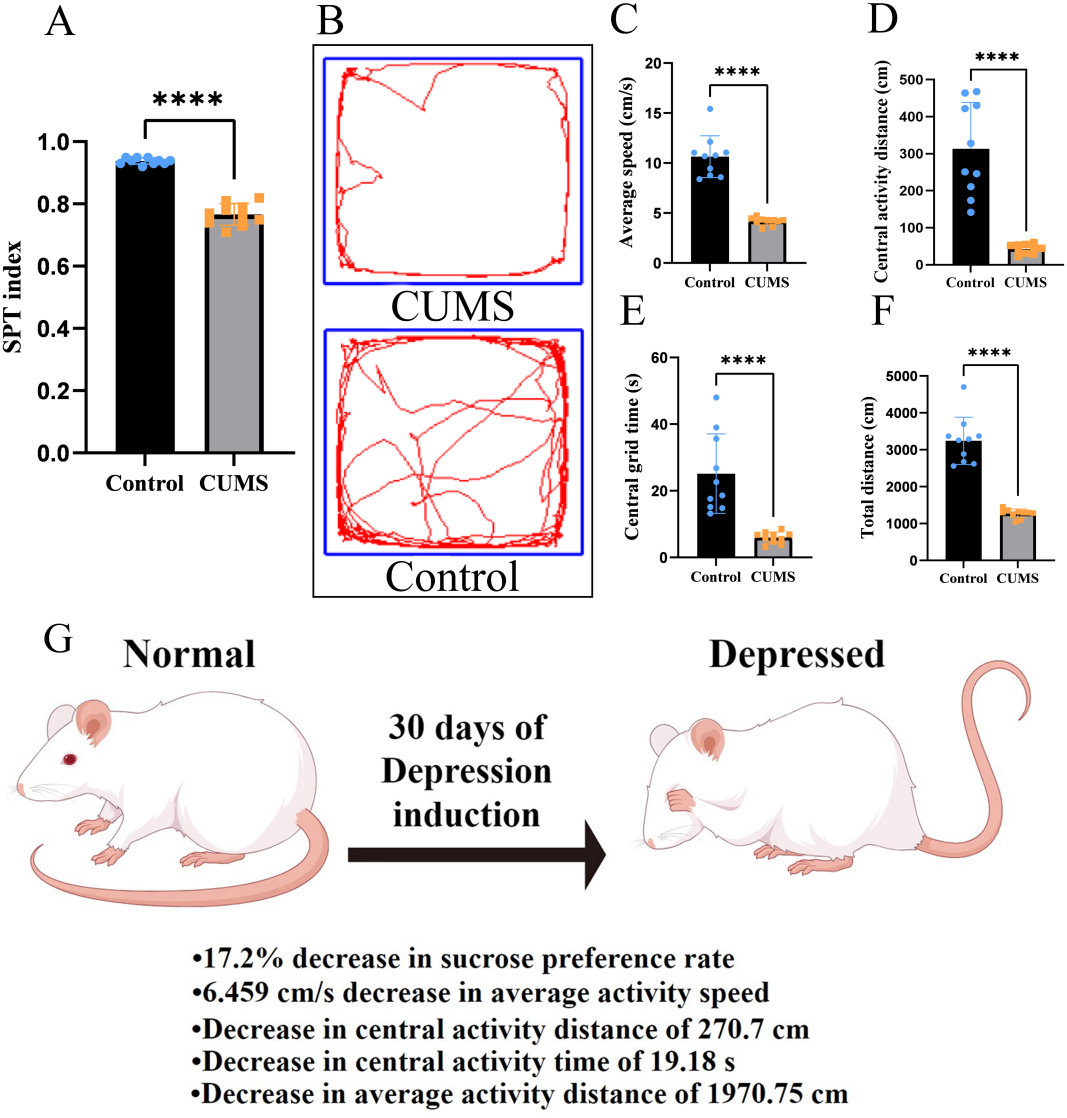
Neurobehavioral evaluation of rats in the CUMS and control groups. (A) Sucrose preference index. (B) Representative images of locomotor trajectories of rats in the control and CUMS groups in the open field test are shown. (C) Total distance traveled, (D) number of entries into the central grid, (E) average velocity, and (F) distance moved within the central zone are presented. *n* = 10. ^∗∗∗∗^*P* < 0.001 *vs.* the control group. CUMS, chronic unpredictable mild stress. (G) Schematic representation of the chronic unpredictable mild stress (CUMS) protocol used to induce depression-like behaviors in rats. A 30-day stress paradigm was applied, leading to notable changes in behavioral performance compared to control rats. Created using Figdraw.

Compared with control counterparts, rats in the CUMS group displayed significantly lower sucrose preference index (mean difference = −0.172, 95% CI [−0.196 to −0.148], *P* < 0.05; Cohen’s *d* = 6.70; Fig. 2A), indicating anhedonia, a core depressive-like behavior. In the OFT evaluations, CUMS rats showed statistically reduced total distance travelled (mean difference = −1,970 cm, 95% CI [−2,402 to −1,538], *P* < 0.05; Cohen’s *d* = 4.29) and average velocity (mean difference = −6.459 cm/s, 95% CI[−7.875 to −5.043], *P* < 0.05; Cohen’s *d* = 4.28), indicating hypo locomotion. Furthermore, they exhibited a decreased time spent in the central zone (mean difference = −19.18s, 95% CI [−27.19 to −11.17], *P* < 0.05; Cohen’s *d* = 3.06) and distance moved within the center area (mean difference=−270.7 cm, 95% CI [−353.9 to −187.5], *P* < 0.05; Cohen’s *d* = 2.25), reflecting increased anxiety-like behavior (Figs. 2B–2F). The above alterations in the behavioral profile were consistent with the successful establishment of the CUMS model (Fig. 2G).

### Results of H&E staining analysis

**Figure 3:**
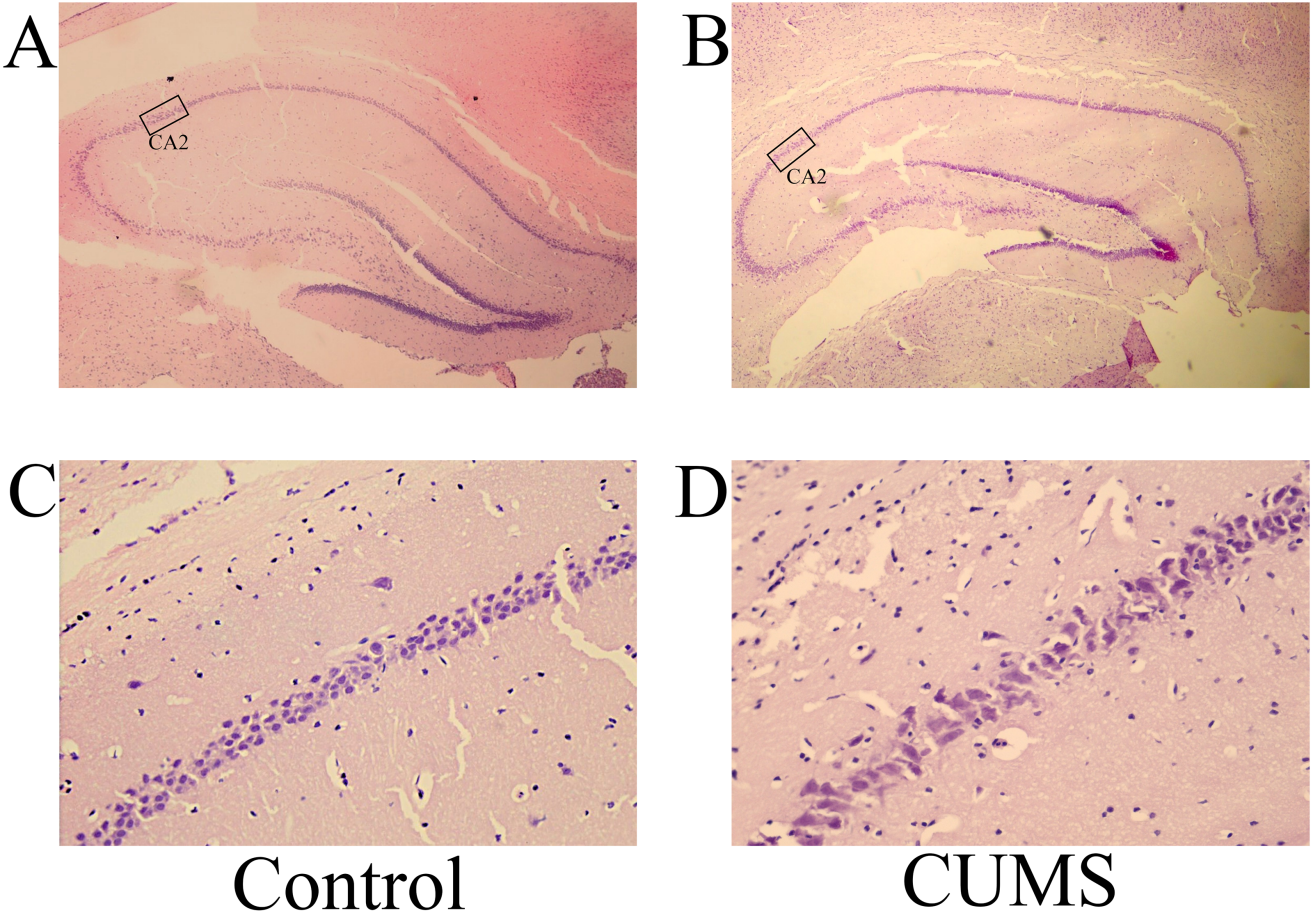
H&E staining of the hippocampal CA2 zone of rats in the CUMS and control groups. Representative images of H&E staining of the CA2 region in the (A) control and (B) CUMS groups at magnification of x200 are shown. H&E staining of CA2 region in the (C) control and (D) CUMS groups at magnification of x400 is presented. (A) and Fig. 3 (A) originate from the same field of view of the same section (Control). (B) and Fig. 3 (B) originate from the same field of view of the same section (CUMS). H&E, hematoxylin and eosin; CUMS, chronic unpredictable mild stress.

**Figure 4:**
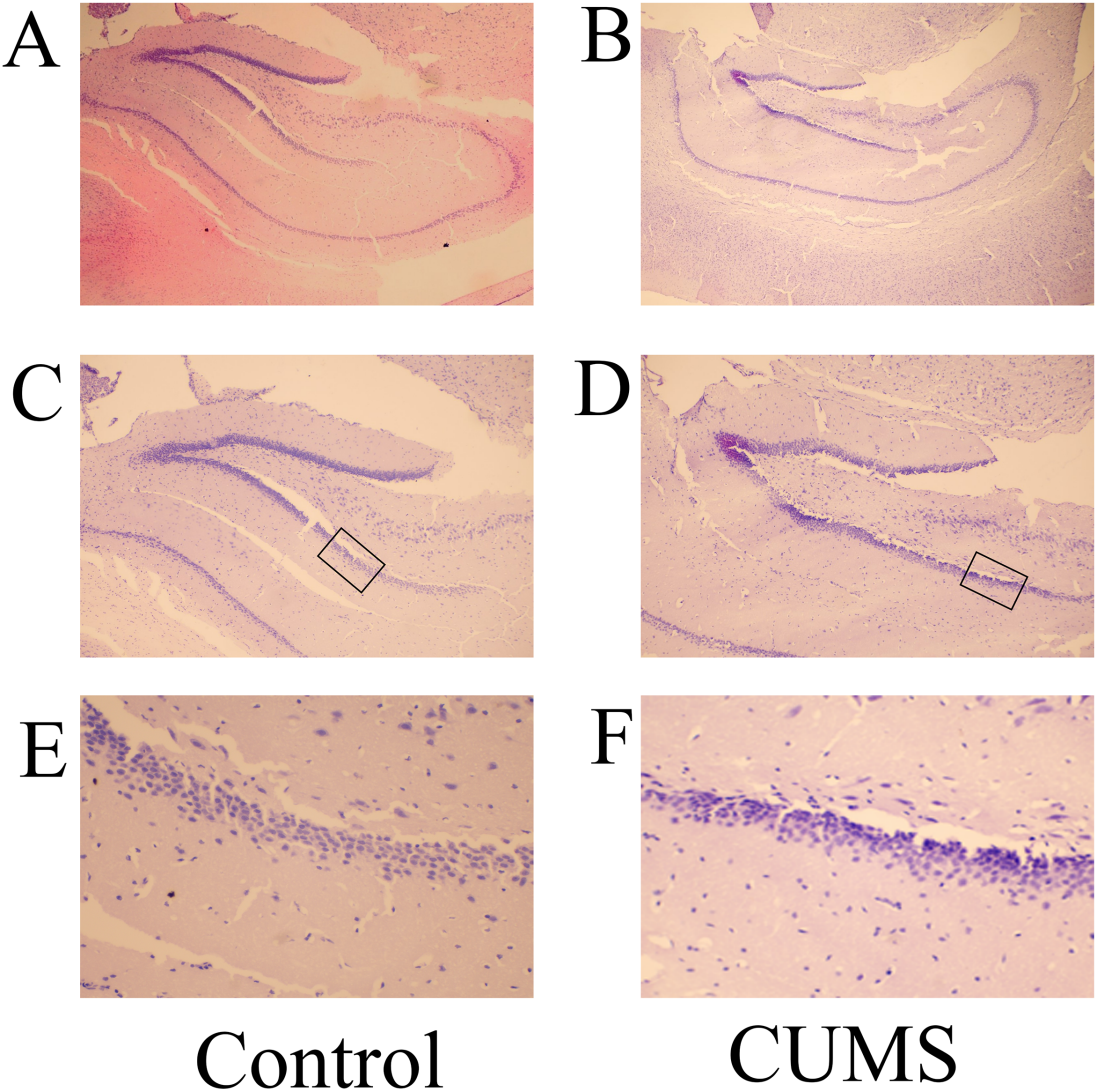
H&E staining of the hippocampal DG zone of rats in the CUMS and control groups. Representative images of H&E staining of the DG region in the (A) control and (B) CUMS groups at magnification of x100 are shown. H&E staining of the DG region in the (C) control and (D) CUMS group at magnification of x200 is presented. H&E staining of DG zone in the (E) control and (F) CUMS groups at x400 magnification. (A) and Fig. 2 (A) originate from the same field of view of the same section (Control). (B) and Fig. 2 (B) originate from the same field of view of the same section (CUMS). H&E, hematoxylingroup, ×200. (E, F) HE staining of DG in control group and eosin; CUMS, chronic unpredictable mild stress.

Microscopic evaluation revealed notable cytoarchitectural differences in the hippocampal CA2 and dentate gyrus (DG) zones between the experimental groups. In the control group, neurons exhibited organized alignment, elevated cellular density, and intact morphology with structurally clearly defined nuclei. By contrast, the brain tissues in the CUMS group displayed disrupted neuronal arrangement, reduced cell density, sparse spatial distribution, and evidence of nuclear fragmentation or atrophy (Figs. 3 and 4). This qualitative observation of reduced cellularity was objectively confirmed by quantifying the density of normal neurons in representative high-power fields. The measurements demonstrated a substantial decrease in the CUMS group, with values falling to 0.242 (CA2) and 0.279 (DG) from control levels of 0.465 (CA2) and 0.611 (DG). These quantitative data, derived from representative sections, offer concrete support for the morphological deficits observed in the CUMS model. It should be noted that this quantification was not based on a full stereological design with serial sections.

### Immunoblotting results of Wnt7a, β-catenin, GSK-3β, and p-GSK-3β in rat hippocampus

**Figure 5:**
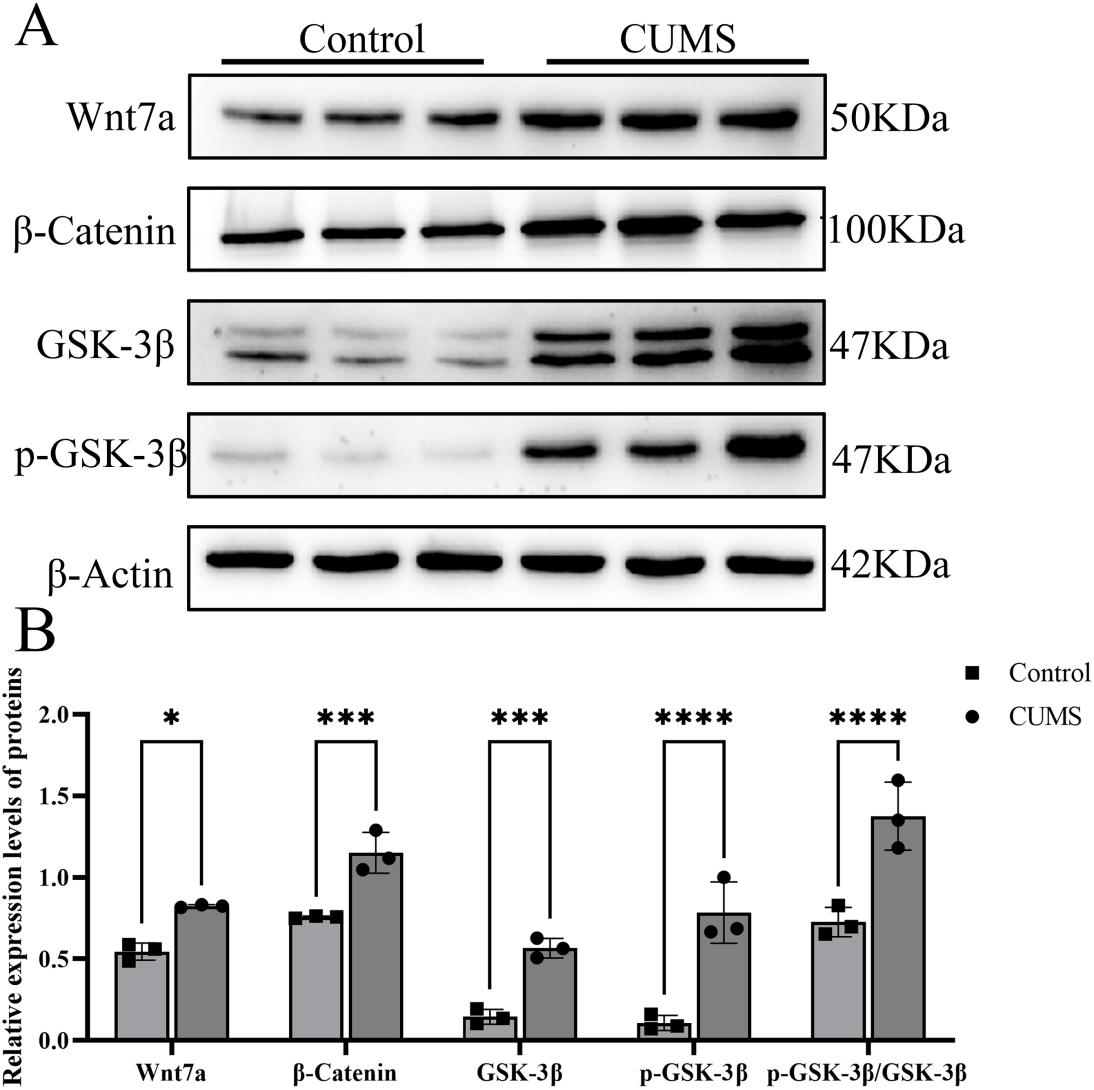
Western blot analysis of Wnt7a, β-catenin, GSK-3β, and p-GSK-3β protein expression in chronic unpredictable mild stress-induced depressed rats. Western blot analysis of Wnt7a, β-catenin, GSK-3β, and p-GSK-3β protein expression in chronic unpredictable mild stress-induced depressed rats. (A) The protein expression levels of Wnt7a, β-catenin, GSK-3β, and p-GSK-3β were detected by western blot analysis. Quantitative analysis of the protein (Wnt7a, β-catenin, GSK-3β, and p-GSK-3β) expression levels and the p-GSK-3β/GSK-3β ratio. (B) Data were analyzed by two-way ANOVA followed by Šídák’s multiple comparisons test. ^∗^*P* < 0.05, ^∗∗∗^*P* < 0.001, ^∗∗∗∗^*P* < 0.0001 *vs.* the control group. Full-length blots are provided in Fig. S1.

Quantitative evaluation of Western blot analysis indicated significant upregulation of Wnt7a, β-Catenin, and total GSK-3β in CUMS rats relative to controls (*P* < 0.05; Fig. 5A). An increase in the phosphorylation level of GSK-3β at Ser9 (p-GSK-3β) was also observed. Accordingly, the p-GSK-3β/GSK-3β ratio, a key indicator of kinase inactivation, was significantly elevated (*P* < 0.05; Fig. 5B). A two-way ANOVA revealed significant main effects of both treatment (rows factor: F (1,20) = 50.71, *p* < 0.0001) and protein (columns factor: F (4,20) = 40.09, *p* < 0.0001), as well as a significant treatment × protein interaction (F (4,20) = 3.936, *p* = 0.0163). Šídák’s *post-hoc* tests confirmed significant upregulation in the CUMS group for all targets: Wnt7a (mean difference = 0.2789, 95% CI [0.0328–0.5251], Cohen’s *d* = 7.48), β-Catenin (mean difference = 0.3950, 95% CI [0.1489–0.6412], Cohen’s *d* = 4.48), GSK-3β (mean difference = 0.4210, 95% CI [0.1749–0.6672], Cohen’s *d* = 7.90), p-GSK-3β (mean difference = 0.6765, 95% CI [0.4304–0.9227], Cohen’s *d* = 4.94), and the p-GSK-3β/GSK-3β ratio (mean difference = 0.6502, 95% CI [0.4040–0.8963], Cohen’s *d* = 4.05). *Post-hoc* power analysis indicated statistical power >80% for all comparisons (α = 0.05). Collectively, these findings suggested that chronic stress is associated with enhanced inhibitory phosphorylation of GSK-3β, suggesting a potential suppression of its kinase activity and a possible activation of the Wnt/β-catenin pathway.

### Immunofluorescence analysis of Wnt7a and β-catenin

**Figure 6:**
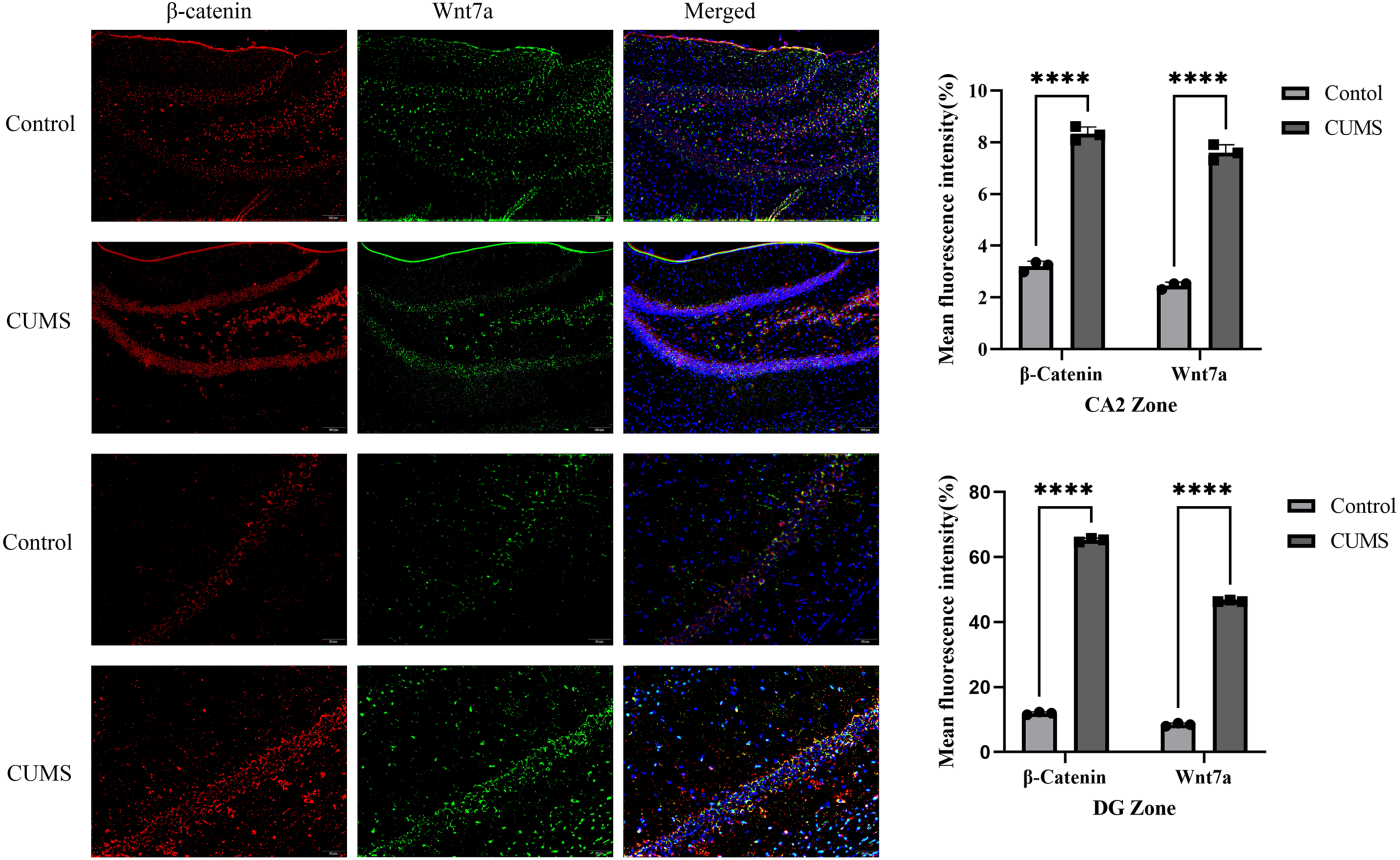
Subcellular localization patterns of Wnt7a and β-catenin in CUMS-induced rats. Subcellular localization patterns of Wnt7a and β-catenin in CUMS-induced rats. Representative immunofluorescence images showing the subcellular localization of β-catenin (red signal), Wnt7a (green signal), and DAPI-stained nuclei (blue). Scale bar, 50 µm. Quantitative analysis revealed that mean fluorescence intensity was significantly elevated for both proteins in the CUMS group compared with the control group. The results are expressed as the mean ± SD (*n* = 3 per group). Statistical significance was determined using two-way ANOVA followed by Šidák’s multiple comparisons test. ^∗∗∗∗^ *p* < 0.0001 *vs.* the control group. CUMS, chronic unpredictable mild stress.

Immunofluorescence staining revealed that both Wnt7a and β-catenin were predominantly localized in the cytoplasm of hippocampal neurons. Quantitative analysis using two-way ANOVA demonstrated significantly elevated fluorescence intensity in CUMS rats and revealed distinct region-specific statistical patterns. In the CA2 region, a significant main effect of treatment was observed (F(1,8) = 29.73, *p* = 0.0006), with no significant treatment × protein interaction (F(1,8) = 0.0069, *p* = 0.9357), indicating a similar and substantial upregulation for both Wnt7a (mean difference = 5.143%, 95% CI [4.623%–5.663%], Cohen’s *d* = 22.3) and β-catenin (mean difference = 5.121%, 95% CI [4.600%–5.641%], Cohen’s *d* = 21.9). In the dentate gyrus (DG), a highly significant interaction was found (F(1,8) = 883.0, *p* < 0.0001), reflecting a differential upregulation where β-catenin increased more profoundly (mean difference = 53.31%, 95% CI [52.31%–54.31%], Cohen’s *d* = 106.2) than Wnt7a (mean difference = 38.03%, 95% CI [37.03%–39.03%], Cohen’s *d* = 99.9). Together, these findings were consistent with the western blot results, supporting the conclusion that protein expression of Wnt7a and β-catenin in hippocampal tissues of rats in CUMS group *versus* with control group (Fig. 6).

## Discussion

This study was designed to investigate the association between depression-like phenotypes and Wnt/β-catenin signaling cascade within hippocampal circuitry using a CUMS-induced rodent model. A CUMS protocol was employed to establish the rodent depression model, facilitating systematic assessment of Wnt/β-catenin pathway dynamics. Our results showed that depressive phenotypes were associated with elevated hippocampal Wnt7a, β-catenin, and GSK-3β protein levels, and enhanced phosphorylation of GSK-3β. Notably, the marked increase in the p-GSK-3β/GSK-3β ratio, which is associated with kinase inhibition, correlates with activation of the Wnt/β-catenin pathway. Collectively, these results describe a correlation between depression-like phenotypes and the activation of the hippocampal Wnt/β-catenin pathway in the CUMS model. The model, established following widely recognized protocols (Nollet, 2021; Mineur, Belzung & Crusio, 2006), was validated by established behavioral tests (OFT and SPT) (Katz, Roth & Carroll, 1981; Tyagi et al., 2025; Duman, 2010; Zhou et al., 2023), which suggested the expected depressive-like phenotypes. CUMS rats exhibited decreased sucrose preference relative to controls, indicating anhedonia, suggesting depressive-like behavior. Notably, no pre-modeling differences were observed between groups, whereas CUMS-exposed rats exhibited significantly impaired performance in SPT and OFT compared to controls. Histological analysis revealed that hippocampal cells displayed typical morphological features associated with depression, including sparse cellular arrangement, pyknosis, fewer protrusions, and reduced density. These features, in combination with the results of behavioral tests, verified the successful establishment of the CUMS depression model.

The canonical Wnt signaling pathway plays a crucial role in neural development, with its evolutionarily conserved molecular cascade widely involved in regulating neuronal differentiation and synaptic network assembly (Zhou et al., 2023). Dysregulation of this pathway has been increasingly associated with various neuropathological manifestations (Gao et al., 2021; Qing et al., 2025), and numerous animal studies suggest that mood stabilizers and antidepressants may enhance neural plasticity by modulating Wnt signaling (Tao et al., 2023; Xiao et al., 2021). Our study observed an association between altered activation of the Wnt/β-catenin signaling pathway in the CUMS depression model and depressive-like behaviors. The functional interpretation of this association, however, is not yet clear. While one perspective might view this activation as contributing to depressive pathogenesis, an equally plausible alternative interpretation is that it represents a compensatory, neuroprotective response to chronic stress, aimed at preserving neuronal structural plasticity and synaptic homeostasis. This latter interpretation is consistent with the well-documented role of Wnt/β-catenin signaling in promoting neuronal survival and repair. Consistent with this canonical activation mechanism, our experimental observations suggest that hippocampal Wnt7a expression is significantly elevated in CUMS depression model rats, with increased total β-catenin protein levels consistent with enhanced GSK-3β Ser9 phosphorylation. Although total GSK-3β levels are elevated, its inhibitory phosphorylation modification remains dominant, suggesting that substantial inhibition of kinase activity is a key mechanism enabling β-catenin accumulation and function. Given that GSK-3β, as a widely expressed kinase, also regulates tau protein, glycogen metabolism, apoptosis, and other related pathways, its aberrant activity is closely associated with neurodegenerative diseases and cell death processes (Hui et al., 2018). The results of this study suggest that activation of the Wnt/β-catenin pathway—particularly the inhibition of GSK-3β kinase activity reflected by its enhanced phosphorylation at Ser9 under CUMS-induced depressive conditions, and the consequent nuclear accumulation of β-catenin—may remodel hippocampal synaptic structure and function, and even impact neural circuits related to learning and memory. Thus, the observed pathway activation suggests an endogenous attempt to promote neuroplasticity. However, it remains a subject for future investigation whether such a response is effectively protective or if, under conditions of persistent and severe stress, it could theoretically become maladaptive.

It is also important to note that our findings of upregulation appear to contrast with some reports showing suppressed Wnt/β-catenin signaling in other stress models (Yang, Song & Xu, 2017; Tao et al., 2023). These discrepancies may stem from differences in stress protocols, animal species, or the timing of molecular assessment, highlighting the context-dependent nature of pathway regulation.

We observed that Wnt7a expression was significantly upregulated in the hippocampus tissue of CUMS model rats compared to the control group. Although previous studies suggest that Wnt7a may be involved in synaptogenesis and plasticity maintenance, its specific regulatory mechanisms under pathological conditions remain incompletely understood (Aghaizu et al., 2023). Furthermore, while the Wnt/β-catenin signaling pathway is thought to potentially regulate adult hippocampal neurogenesis—particularly the maturation and integration of newborn neurons—the functional significance of this role in the context of depression is still open to multiple interpretations. For instance, although studies have shown that Wnt7a can promote the aggregation of synaptic proteins and the expansion of growth cones (Anand et al., 2023; Piers et al., 2024), its net effect within the complex *in vivo* micro-environment requires further validation. Based on the current findings, it is hypothesized that during depression, a subset of neurons may attempt to stimulate dendritic branching and counteract widespread dendritic atrophy by up-regulating Wnt7a and activating the Wnt/β-catenin pathway. This aligns with the view of a potential compensatory mechanism. However, whether this response truly represents an effective compensatory mechanism or is merely an epiphenomenon of the pathological process remains elusive. The causal relationship between this neuroplastic response and the onset of depression, as well as its specific contribution, needs to be clarified through more targeted functional experiments—such as precise regulation of specific cell types, or intervention studies within specific time windows.

Together, the robust effect sizes observed (Cohen’s d ranging from 4.5 to 106.2) provide strong quantitative support for the conclusion that CUMS may induce a marked activation of the hippocampal Wnt/β-catenin pathway, notwithstanding the modest sample size for molecular analyses.

In the present study, we observed characteristic indicators of Wnt/β-catenin signaling pathway activation in the CUMS depression model (such as enhanced phosphorylation of GSK-3β at Ser9 and β-catenin accumulation). These data establish a correlation and describe a molecular phenomenon within a pathological state. The current data are insufficient to conclusively confirm whether this endogenous Wnt signaling activation is a causative factor in depression or a compensatory effort of the organism, nor can they establish its active involvement in triggering intrinsic antidepressant effects. Although preclinical studies suggest that certain antidepressant drugs may enhance neural plasticity by modulating the Wnt pathway, the functional output of this pathway in a complex pathological milieu—whether it ultimately promotes recovery or exacerbates damage—is likely highly context-dependent and involves intricate intrapathway crosstalk. Given the pathway’s well-known role in neurogenesis and synaptic homeostasis, the hypothesis of its involvement in depressive pathology is mechanistically plausible. However, validating its exact causal role and therapeutic potential requires functional evidence from targeted intervention experiments, such as hippocampus-specific Wnt7a gene knockout in rats or pharmacological regulation of Wnt pathway components. These interventional approaches represent critical future directions not addressed in the present study.

Although traditional experimental methods can provide essential data, they can be limited in exploring the molecular mechanisms underlying complex diseases, such as depression. Artificial intelligence (AI) offers a powerful complementary approach. For example, machine learning and deep learning algorithms can efficiently integrate and analyze multi-omics data, including transcriptomics, proteomics, and metabolomics, potentially uncovering complex interaction networks and identifying potential biomolecular markers related to the Wnt/β-catenin signaling and other depression-related pathways, such as the neurotrophic factor and neuroinflammation pathways. Recently demonstrated in AI-driven neuroimaging studies of major depressive disorder (Chen et al., 2025), AI-driven computer vision technology also holds promise for enabling more refined, high-throughput, and automated analysis of animal behavioral data, such as that generated by tests like the sucrose preference test (SPT) employed in this study. This potential is validated by its successful application in rodent depression models using deep learning-based behavioral phenotyping (Tseng et al., 2023). This approach can enable the detection of subtle behavioral manifestations that can be overlooked by traditional experimental methods, thus allowing for more precise associations between these behaviors and particular molecular changes, such as alterations in Wnt/β-catenin expression levels. The application of computational biology approaches has increasingly expanded into more disease research fields, including the development of clinical decision-making systems for osteosarcoma based on deep belief networks to improve diagnostic and therapeutic efficiency (Li et al., 2022a), as well as machine learning models designed to anticipate the risk of lymph node transfers in Ewing’s sarcoma, thus guiding clinical practice (Li et al., 2022b). Future research on depression could benefit from these cross-disciplinary success stories, combining AI-based computational models, such as network pharmacology and knowledge graph modeling, with experimental findings, like ours. Such integration could enable more systematic simulation and prediction of the dynamic effect of the Wnt/β-catenin pathway in the onset and progression of depression, thus providing novel insights into its potential as a therapeutic target.

## Conclusion

In summary, this study utilized the CUMS rat depression model and observed indications of a significant activation of the Wnt/β-catenin signaling pathway in the hippocampus under depressive-like conditions. The upregulation of Wnt7a and β-catenin, along with the elevated p-GSK-3β/GSK-3β ratio, is associated with the depressive phenotype. These findings contribute to the understanding of depression pathogenesis and suggest key components of the Wnt/β-catenin pathway for further investigation as potential therapeutic targets.

## Limitations

The interpretation of our findings should be considered in the context of several methodological limitations. These limitations primarily concern sample size, the correlational nature of the evidence, and the scope of the experimental model, as detailed in the following sections.

### Sample size and statistical considerations

The sample size for the Western blot and immunofluorescence analyses was modest (*n* = 3 per group). Although *post-hoc* power analysis indicated sufficient statistical power (>80%) for the observed effects, this small sample may affect the generalizability of the results and necessitates validation in larger cohorts.

### Lack of pharmacological or genetic controls

Our study did not include positive or negative control interventions (*e.g.*, lithium treatment or other known Wnt pathway modulators). This omission precludes a direct functional validation of pathway activation status and means that while our data are consistent with pathway activation, we cannot definitively rule out other off-target effects of the CUMS procedure.

### Limitations in pathway activation markers

The assessment of Wnt/β-catenin pathway activation relied primarily on the increased p-GSK-3β/GSK-3β ratio and elevated β-catenin levels. We did not obtain direct evidence of β-catenin nuclear translocation or measure the expression of its canonical downstream target genes (*e.g.*, Axin2, BDNF), which would provide more definitive proof of pathway engagement.

### Correlational nature of the evidence

The observational design of this study, without complementary gain- or loss-of-function experiments, establishes a correlation but does not permit causal inference regarding the role of the Wnt/β-catenin pathway in the depressive-like behaviors observed.

### Lack of cellular and regional specificity

While immunofluorescence revealed increased Wnt7a and β-catenin within the hippocampal formation, we did not perform co-staining with specific neuronal or glial markers (*e.g.*, NeuN, GFAP) or conduct separate analyses for distinct hippocampal subregions (*e.g.*, CA1, CA3, DG). Consequently, the precise cellular subtypes and anatomical loci exhibiting altered expression remain unidentified, limiting mechanistic interpretation at the cellular level.

### Single time point and sex limitation

This research was conducted exclusively in male rats at a single post-CUMS time point. Therefore, our study cannot address potential sex differences in the response or elucidate the temporal dynamics of pathway activation and its relationship to behavioral changes over time.

### Future directions

Studies designed to address these limitations—including cell-type-specific investigations, incorporation of pharmacological/genetic controls, direct assessment of β-catenin transcriptional activity, and examinations across sexes and time points—will be crucial to build upon these initial correlative findings.

## Supplemental Information

10.7717/peerj.20837/supp-1 Supplemental Information 1 Uncroped WB membrane

10.7717/peerj.20837/supp-2 Supplemental Information 2 Author checklist

10.7717/peerj.20837/supp-3 Supplemental Information 3 The role and mechanisms of the Wnt7a/β-catenin signalling pathway in depression

10.7717/peerj.20837/supp-4 Supplemental Information 4 Raw statistical data for analysis

10.7717/peerj.20837/supp-5 Supplemental Information 5 G*Power analysis

10.7717/peerj.20837/supp-6 Supplemental Information 6 Raw data of WB analysis

10.7717/peerj.20837/supp-7 Supplemental Information 7 IF raw data for analysis

10.7717/peerj.20837/supp-8 Supplemental Information 8 Raw data of IF analysis

10.7717/peerj.20837/supp-9 Supplemental Information 9 Original IF channel-separated image

10.7717/peerj.20837/supp-10 Supplemental Information 10 HE-stained cell density count

10.7717/peerj.20837/supp-11 Supplemental Information 11 Raw data of OFT

10.7717/peerj.20837/supp-12 Supplemental Information 12 Raw data of SPT

## Additional Information and Declarations

### Data Availability

The following information was supplied regarding data availability: The raw data is available in the Supplemental Files.
