# Supplementary material for: Hippocampal expression of Wnt7a and β-catenin in depression: evidence from chronic unpredictable mild stress
Source: PeerJ. 2026 Feb 19;14:e20837. doi: 10.7717/peerj.20837 (PMC12925407; doi:10.7717/peerj.20837)
Supplement: Supplemental Information 1 [file peerj-14-20837-s001.docx]

**The supplementary images below correspond to the uncropped Wersten-Blot images in Figure 5.**

**Wnt7a**

The raw, uncropped image captured, with the molecular weight ladder shown on the left and the observed molecular weight of Wnt7a on the right; the boxed section represents the portion used for analysis. The boxed region indicates the section used for analysis, with lane labels displayed above.


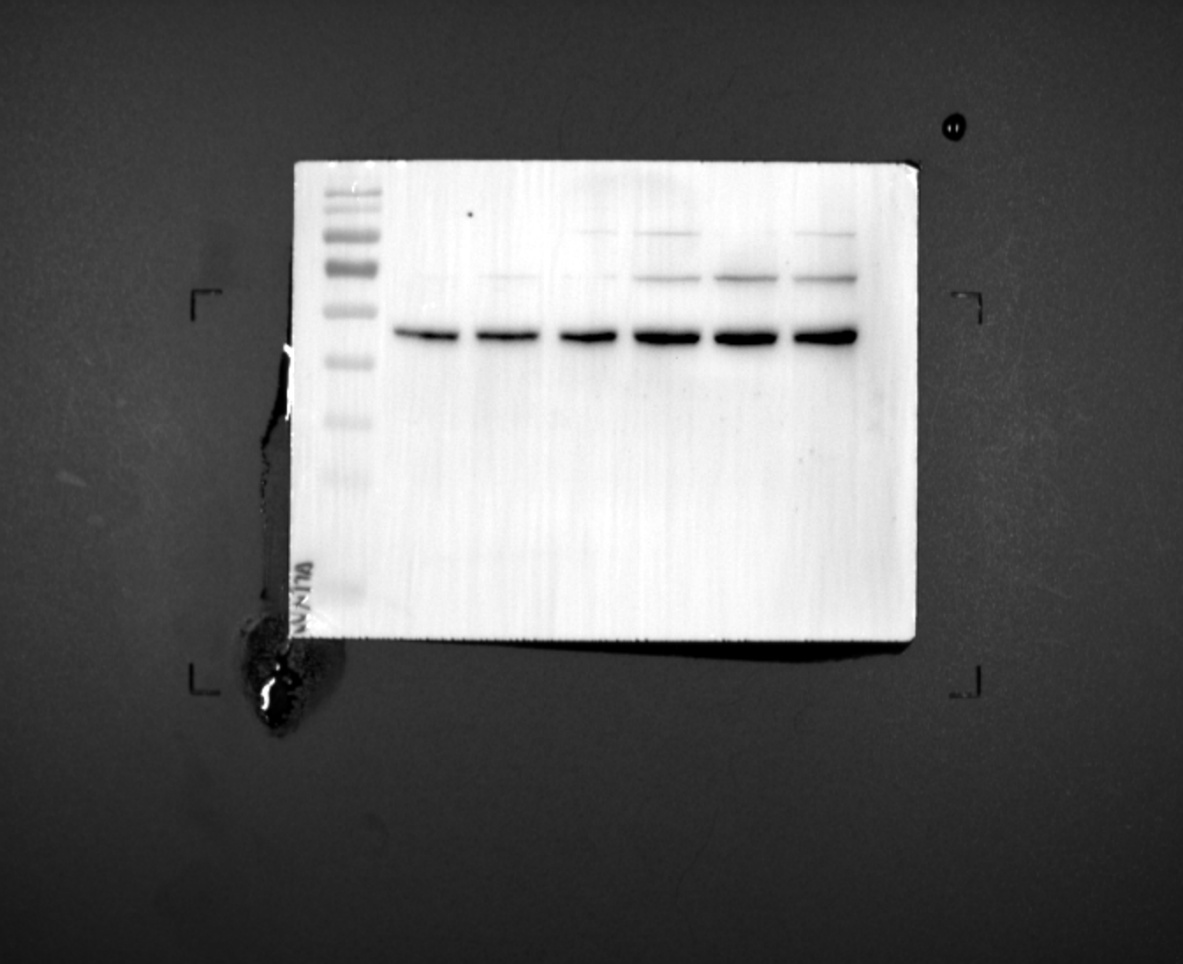


**50KDa**

CUMS

CUMS

CUMS

Control

Control

Control

**β-Catenin**

The raw, uncropped image captured, with the molecular weight ladder shown on the left and the observed molecular weight of β-Catenin on the right; the boxed section represents the portion used for analysis. The boxed region indicates the section used for analysis, with lane labels displayed above.


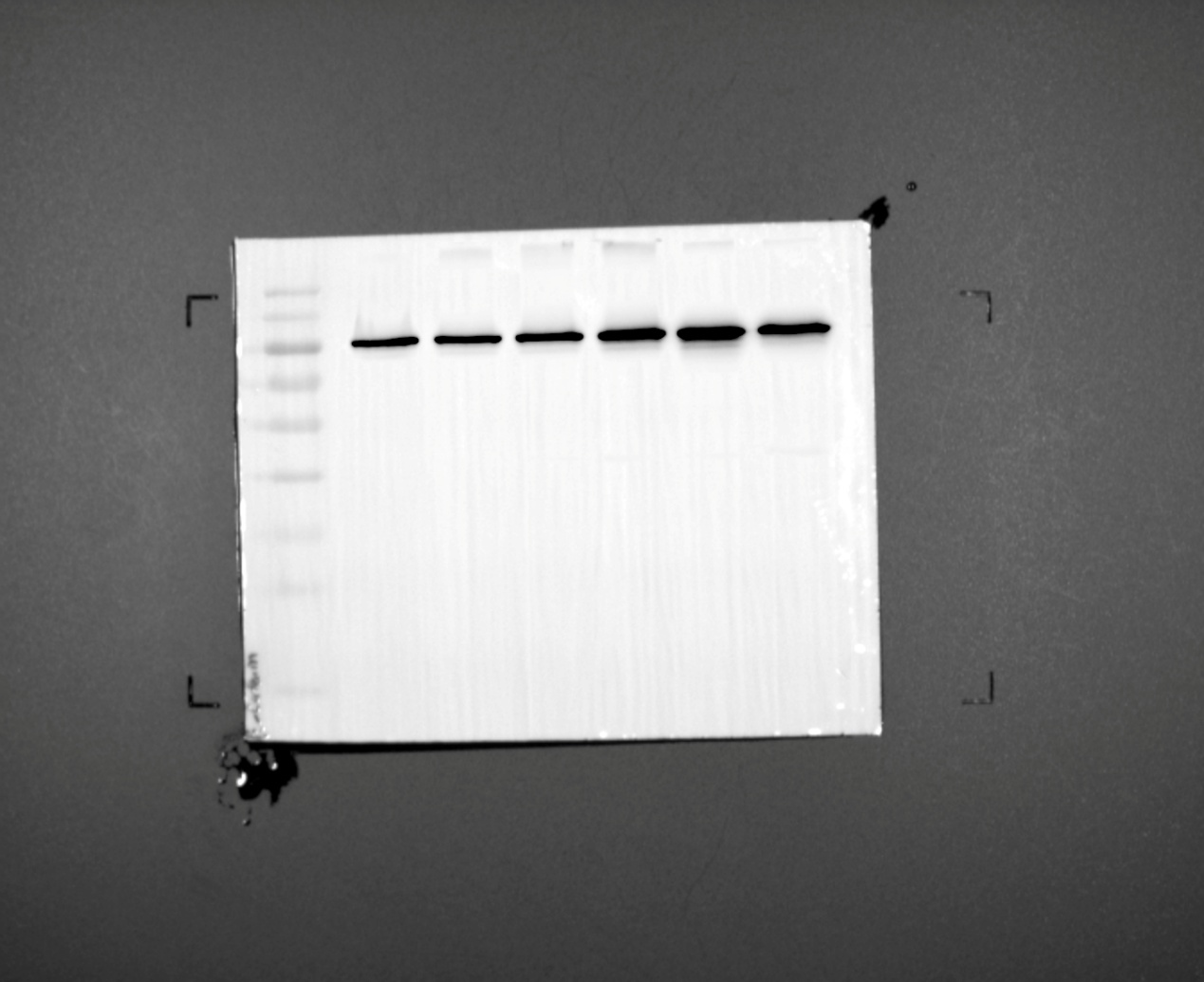


CUMS

CUMS

CUMS

Control

Control

Control

**100KDa**

**GSK-3β**

The raw, uncropped image captured, with the molecular weight ladder shown on the left and the observed molecular weight of GSK-3β on the right; the boxed section represents the portion used for analysis. The boxed region indicates the section used for analysis, with lane labels displayed above.


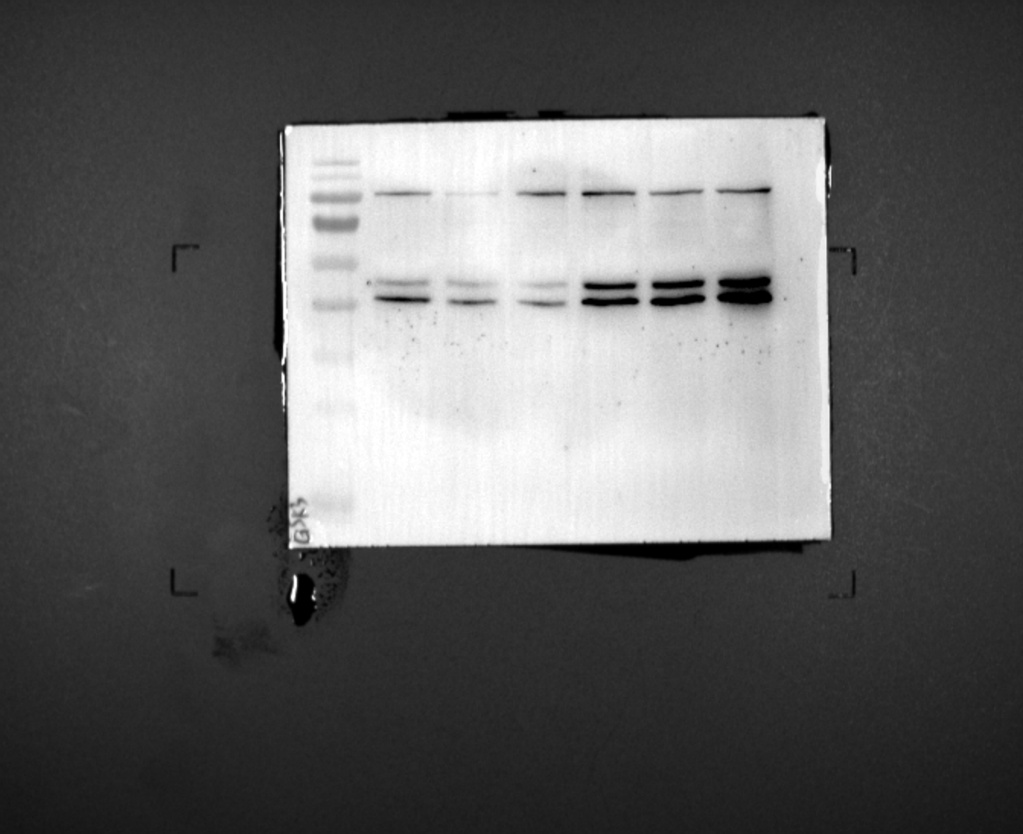


**47KDa**

CUMS

CUMS

CUMS

Control

Control

Control

**p-GSK-3β**

The raw, uncropped image captured, with the molecular weight ladder shown on the left and the observed molecular weight of p-GSK-3β on the right; the boxed section represents the portion used for analysis. The boxed region indicates the section used for analysis, with lane labels displayed above.


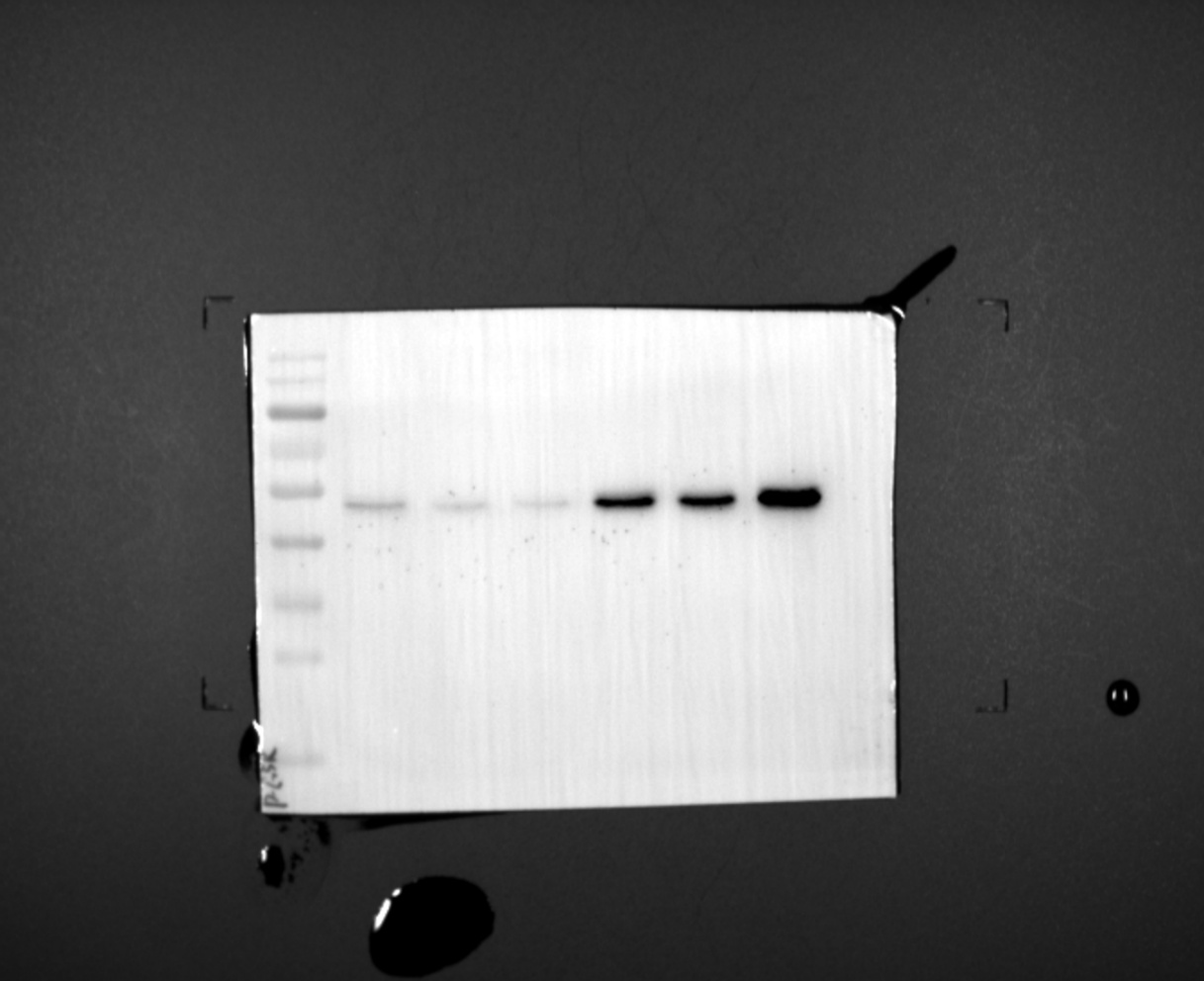


**47KDa**

CUMS

CUMS

CUMS

Control

Control

Control

**β-actin**

The raw, uncropped image captured, with the molecular weight ladder shown on the left and the observed molecular weight of β-actin on the right; the boxed section represents the portion used for analysis. The boxed region indicates the section used for analysis, with lane labels displayed above.


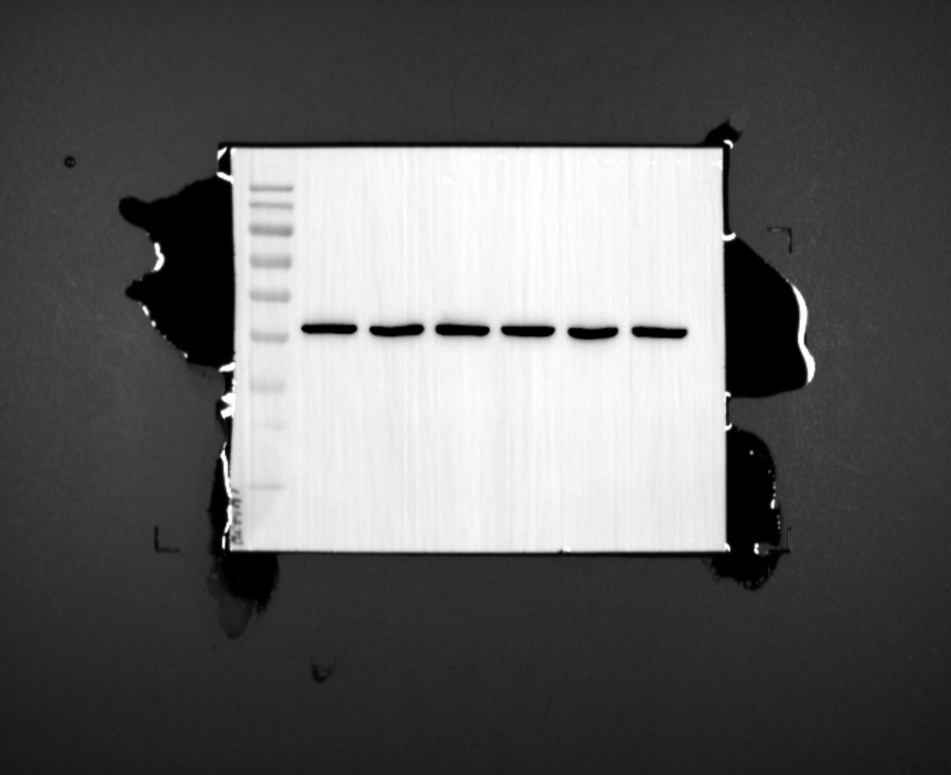


**42KDa**

CUMS

CUMS

CUMS

Control

Control

Control
