# Supplementary material for: Hippocampal expression of Wnt7a and β-catenin in depression: evidence from chronic unpredictable mild stress
Source: PeerJ. 2026 Feb 19;14:e20837. doi: 10.7717/peerj.20837 (PMC12925407; doi:10.7717/peerj.20837)
Supplement: Supplemental Information 3 [file peerj-14-20837-s003.docx]

**Research protocol**

**Abstract**

Depression will become the most significant cause of disability in humans, but its pathogenesis remains unclear. Research has shown that abnormalities in the volume and structure of the hippocampus are one of its typical symptoms. Wnt7a plays multiple roles in adult hippocampal neurogenesis by regulating the development of new neurons. The Wnt/β-catenin signaling pathway is a key regulator of cell proliferation. We hypothesise that the onset of depression is associated with abnormalities in the Wnt/β-catenin signaling pathway. In this study, we established a depression model using chronic unpredictable mild stress and observed changes in hippocampal neural structure using techniques such as light microscopy; We employed Western blot and immunofluorescence techniques to detect the key protein Wnt7a in the Wnt/β-catenin signalling pathway in the hippocampus, observing its effects on the signalling pathway. This study aims to preliminarily explore the potential relationship between hippocampal volume abnormalities and the Wnt/β-catenin signalling pathway, providing experimental evidence to support further research into the pathogenesis of depression.

**Basis for project initiation**

**Purpose of the study**

Depression is a chronic, recurrent affective disorder characterised by high rates of disability and suicide [1-2]. It significantly impacts mental and physical health and imposes a substantial burden on society. By 2030, depression is projected to become the leading cause of global disease burden [3]. Research into the pathogenesis and prevention of depression is increasingly important, yet its underlying mechanisms remain incompletely understood. This study employs a chronic unpredictable mild stress (CUMS) model to induce depression in rats, using haematoxylin and eosin (HE) staining and Nissl staining to observe changes in neuronal morphology and structure. Western blot analysis is used to detect the expression levels of key proteins in the Wnt/β-catenin signalling pathway. This study aims to preliminarily explore the relationship between hippocampal volume abnormalities in the depression pathogenesis process and the Wnt/β-catenin signalling pathway, thereby providing experimental evidence for further research into the pathogenesis of depression.

**Research content**

**(1) Establishing a depression animal model and conducting behavioural testing**

**1) Establishing a depression animal model**

The study used SPF-grade SD adult male rats, which were acclimatised for one week before being randomly divided into a control group and a model group. The control group received no special treatment and was maintained under normal conditions. The model group rats were subjected to chronic unpredictable mild stress (CUMS) using the classic modelling method, with 10 different chronic stressors administered over a 30-day period. These included: 24-hour wet bedding (500 ml of tap water poured into the rat cage), 24-hour fasting, 24-hour water deprivation, 24-hour behavioural restraint stress, 5-minute cold water swimming stress at 4°C, low-current foot shock stress, 24-hour ultrasonic repellent, 24-hour cage tilting at 45°, 1-minute tail pinching, and 15-minute cage shaking.

The above stimuli were repeated three times, with the order randomised but the same stressor not appearing consecutively, to prevent the rats from anticipating the stimuli.

**2)behavioural testing**

**Sucrose Preference Test**

Conducted at 9:00 AM on day 30, all experimental animals were deprived of water for 12 hours prior to the experiment. Two bottles were placed in the mouse cage: one containing 200 ml of tap water and the other containing 200 ml of 1% sucrose solution. Before the experiment, the water bottles were checked for leaks. After 1 hour, the consumption of sucrose solution and tap water was measured, and the sucrose preference rate was calculated. Calculate using the following formula:

Sucrose preference rate (%) = Sucrose consumption (ml) / Total consumption of sucrose and tap water (ml) × 100%

**Open field test**

The test area is 120 cm long, 90 cm wide, and 35 cm high, with a black bottom. The test is conducted in a relatively quiet room at the same time each day. The rat is gently placed in the central square by its tail, and then tested using the Mouse Doctor video analysis system. The test includes total walking distance, time spent in the central area, number of times standing, and grooming behaviour. After each rat is tested, the open field must be thoroughly cleaned with 75% alcohol to remove faeces and urine. The experiment can only continue after the alcohol has evaporated and the area is clean.

**(2) Morphological analysis of hippocampal neuronal structure in animals**

**Animal grouping and treatment:**

Rats were divided into: ① Control group: no special treatment

② Model group: given CUMS

Rats in both groups were euthanised after the model was completed for use in the experiment.

Structural observation: After perfusion fixation of the hearts of rats in both groups, brain tissue was extracted, dehydrated, embedded, and sectioned. HE staining or Nissl staining was used to observe morphological changes in hippocampal neurons in both groups of rats.

**(3) Western Blot (WB) Experimental Procedure:**

To detect the protein expression levels of Wnt7a, β-catenin, GSK-3β, and p-GSK-3β in hippocampal tissues, Western blot analysis will be performed. The procedure is as follows: hippocampal tissues from control and CUMS model rats will be homogenized in RIPA lysis buffer to extract total protein, which will be quantified using a BCA assay. Equal amounts of protein will be separated by SDS-PAGE and transferred onto PVDF membranes. The membranes will then be blocked with 5% skim milk and incubated sequentially with corresponding primary antibodies and HRP-conjugated secondary antibodies. Finally, the protein bands will be visualized using a chemiluminescent substrate, and the band intensities will be quantified using Image J software with β-actin as the internal control.

**(4) Immunofluorescence (IF) Experimental Procedure:**

To observe the expression and localization of Wnt7a and β-catenin proteins in the hippocampal tissues, immunofluorescence staining will be conducted. The procedure is as follows: rats from control and CUMS model groups will be transcardially perfused with fixative, and their brains will be harvested and processed into coronal frozen sections. The sections will be permeabilized, blocked, and then incubated with primary antibodies against Wnt7a and β-catenin, followed by corresponding fluorescent dye-conjugated secondary antibodies. Nuclei will be counterstained with DAPI. Finally, the sections will be observed and imaged under a laser scanning confocal microscope, and the fluorescence intensity in specific hippocampal regions will be quantified using Image J software.

**Current status and development trends of domestic and international research**

**(1) Studying changes in brain tissue structure is one of the key areas of basic research on depression.**

Patients with depression exhibit abnormalities in the classic neuroanatomical circuit structure and function of the limbic system-cortex-striatum-globus pallidus-thalamus pathway, with the most prominent abnormalities being changes in the volume of the hippocampus and amygdala, as well as structural alterations in the frontal and parietal lobes [4]. Among these, the hippocampus is the most extensively studied region in brain tissue research. Whether in depression animal models [5] or depression patients [6-8], research findings consistently show abnormal structural changes in the hippocampus, which manifest behaviourally as impaired learning and memory abilities and reduced motor activity.

**(2) The relationship between depression and hippocampal abnormalities**

Autopsies of deceased depression patients have also confirmed reduced hippocampal volume, decreased numbers and density of glial cells, and atrophy of the neural fibre network composed of neurons and glial cells [9]. Although the exact causes of depression remain unclear, it is certain that prolonged stress or major life events are important triggers for the onset of depression. Prolonged stress can alter the plasticity of hippocampal neurons in experimental animals and reduce the density of dendritic spines. Following depression treatment, the hippocampal volume of depressed patients increased compared to pre-treatment levels [11]. Therefore, depression is not merely a purely functional mental disorder; structural damage in certain brain regions serves as a prerequisite for the organic lesions associated with depression.

**(3) The relationship between the hippocampus and the Wnt/β-catenin signalling pathway in depression**

The Wnt signalling pathway plays a crucial role in embryonic development and neural system development, such as synaptogenesis, dendritic morphology formation, and the development of structures like the hippocampus. The Wnt/β-catenin signalling pathway is also closely associated with neural regeneration in the adult hippocampus. Neural regeneration is crucial in the onset, progression, and treatment of depression, suggesting that the Wnt signalling pathway may be involved in the depressive process [12].

A subclass of Wnt proteins is called Wnt1 proteins, including Wnt1, Wnt2, Wnt3a, Wnt3, Wnt7a, Wnt8b, and Wnt10b, which participate in Wnt/β-catenin signal transduction [13]. A characteristic feature of the Wnt/β-catenin signalling pathway is the accumulation of the adhesion-related protein β-catenin (i.e., β-catenin) in the cytoplasm, followed by its translocation to the nucleus. It then binds to transcription factors such as T-cell factor (TCF) and lymphoid enhancer-binding factor (LEF) to activate target genes. The typical pathway primarily regulates cell proliferation [13].

Researchers both domestically and internationally have conducted in-depth investigations into the causes of hippocampal abnormalities in depression and stress-related disorders. Some studies have shown that Wnt7a plays a role in regulating proliferation and differentiation. In another study, long-term infusion of Wnt7a into the rat hippocampus led to an increase in the number of immature neurons [13]. Additionally, immature neurons in Wnt7a knockout mice have been shown to exhibit reduced dendritic arborisation. These studies suggest that Wnt7a plays multiple roles in adult hippocampal neurogenesis by controlling the developmental process of newly generated neurons. The Wnt/β-catenin signaling pathway is a key pathway regulating cellular functions such as proliferation, migration, differentiation, genetic stability, apoptosis, and stem cell renewal. However, the role of the Wnt/β-catenin signaling pathway mediated by Wnt7a in the pathogenesis of depression remains unclear.

**(4) The role of β-catenin in various neurodegenerative diseases**

Studies have shown that this protein participates in the Wnt/β-catenin signalling pathway during the pathogenesis of epilepsy, thereby contributing to epileptic seizures and neurogenesis induced by hippocampal sclerosis, and may serve as a future therapeutic target for epilepsy [14].

Catenin proteins also participate in the regulation of aerobic glycolysis through the Wnt/β-catenin pathway, and impaired brain glycolytic function contributes to the development of Alzheimer's disease (AD) [15].

In summary: During the pathogenesis of depression, the hippocampus exhibits distinct structural abnormalities, reduced hippocampal volume, decreased numbers and density of glial cells, and atrophy of the neural fibre network composed of neurons and glial cells. The Wnt/β-catenin signalling pathway is one of the key pathways regulating cell proliferation and differentiation, but its role in the development of depression remains unclear, particularly regarding the role of the Wnt/β-catenin signalling pathway mediated by Wnt7a in the pathogenesis of depression. Therefore, we used a depression animal model to detect changes in Wnt7a and β-catenin in the hippocampus, and explored the regulatory role of the Wnt/β-catenin signalling pathway in the onset of depression, with the aim of providing experimental evidence for the study of the pathogenesis of depression.

**Innovation Points and Project Features**

Elucidating the role of the Wnt/β-catenin signalling pathway mediated by Wnt7a in the hippocampus of depressed model rats in the pathogenesis of depression provides experimental evidence for studying the pathogenesis of depression.

**References**

[1] Liang YD, Wang YL, Li Z, et al. Caregiving burden and depression in paid caregivers ofhospitalized patients: a pilot study in china[J]. BMC Public Health, 2017, 18(1):53.

[2] Nikolina Jovanović, Julian Beezhold, Masaru Tateno, et al. Depression and suicidality among

psychiatric residents-results from a multi-country study[J]. J Affect Disord 2019,249:192-198.

[3] Kaj S Christensen, Wenche Haugen, Manjit K Sirpal, et al. Diagnosis of depressed young

people--criterion validity of WHO-5 and HSCL-6 in Denmark and Norway[J]. Fam Pract 2015,

32(3):359-363.

[4] 秦玲娣, 赖丽莎, 陈少琼,邓星河,康庄,单鸿. 抑郁症患者边缘系统-皮层-纹状体-苍白球-丘脑神经环路相关结构的磁共振成像研究. 中华临床医师杂志 (电子版), 2010, 6(3): 15-18.

[5] Mehta V, Singh T R, Udayabanu M. Quercetin ameliorates chronic unpredicted stress-induced behavioral dysfunction in male Swiss albino mice by modulating hippocampal insulin signaling pathway. Physiology & Behavior, 2017, 182:10.

[6] Ahdidan J, Hviid LB, Chakravarty MM. Longitudinal MR study of brain structure and hippocampus volume in major depressive disorder. Acta Psychiatr Scand, 2011, 123(3): 211-219.

[7] Serrano-Sosa M, Sampathgiri K, Spuhler K D, et al. The importance of identifying functional

Val158Met polymorphism in catechol-O-Methyltransferase when assessing MRI-based volumetric measurements in major depressive disorder[J]. Brain imaging and behavior, 2020, 14(6): 2762-2770.

[8] Colle R, Segawa T, Chupin M, Dong M, Hardy P, Falissard B. Early life adversity is associated with a smaller hippocampus in male but not female depressed in-patients: a case–control study. Bmc

Psychiatry, 2017, 17(1):71.

[9] Gitte Nikolajsen. A reduced number of hippocampal granule cells does not associate with an

anhedonia-like phenotype in a rat chronic mild stress model of depression. Stress-The International Journal on the Biology of Stress, 2010, 13(2):95-105.

[10] Qiao H, An S C, Xu C, et al. Role of proBDNF and BDNF in dendritic spine plasticity and

depressive-like behaviors induced by an animal model of depression[J]. Brain research, 2017, 1663:29-37.

[11] Nordanskog P, Dahlstrand U, Larsson M R, Larsson E M, Knutsson L, Johanson A. Increase in hippocampal volume after electroconvulsive therapy in patients with depression: a volumetric magnetic resonance imaging study. Journal of Ect, 2010, 26(1):62-67.

[12] 周文娟.Wnt2和Wnt3在慢性束缚应激引起的抑郁行为中的作用及其机制研究[D].山东大学,2015..

[13] Anand AA, Khan M, V M, Kar D. The Molecular Basis of Wnt/β-Catenin Signaling Pathways in Neurodegenerative Diseases. Int J Cell Biol. 2023 Sep 21;2023:9296092. doi: 10.1155/2023/9296092. PMID: 37780577; PMCID: PMC10539095.

[14] Gautam V, Rawat K, Sandhu A, Kumar A, Kharbanda PS, Medhi B, Bhatia A, Saha L. Exploring the effect of 6-BIO and sulindac in modulation of Wnt/β-catenin signaling pathway in chronic phase of temporal lobe epilepsy. Neuropharmacology. 2024 Apr 2;251:109931. doi: 10.1016/j.neuropharm.2024.109931. Epub ahead of print. PMID: 38570067.

[15] Liu J, Wei AH, Liu TT, Ji XH, Zhang Y, Yan F, Chen MX, Hu JB, Zhou SY, Shi JS, Jin H, Jin F. Icariin ameliorates glycolytic dysfunction in Alzheimer's disease models by activating the Wnt/β-catenin signaling pathway. FEBS J. 2024 Feb 23. doi: 10.1111/febs.17099. Epub ahead of print. PMID: 38400523.

**Technical approach, problems to be solved, and expected results**

**(1) Technical approach**

Experimental SD rats

Adaptive feeding for one week

Control

CUMS

CUMS molding 30d

Sucrose preference test

Open field test

Behavioral testing

Separate the hippocampi of the two groups of rats.

Western blot analysis

HE staining, immunofluorescence

Detection of hippocampal Wnt7a, β-catenin, GSK-3β, and p-GSK-3β expression levels

Observation of changes in the structure of neurons in the hippocampus of rats

Comparative analysis of structural changes in rat hippocampal neurons before and after modelling, detection of Wnt7a, β-catenin, GSK-3β, and p-GSK-3β expression levels in the hippocampus, investigation of the Wnt7a/β-catenin signalling pathway, and exploration of the pathogenesis of depression.

**(2) Problems to be addressed**

We will use a depression animal model to measure the expression levels of Wnt7a and β-catenin in the hippocampus, thereby preliminarily elucidating the regulatory role of the Wnt/β-catenin pathway in the pathogenesis of depression, with the aim of providing experimental evidence for studying the mechanisms underlying depression.

**(3) Expected outcomes**

1) The behavioural effects of Wnt7a on CUMs depression model rats.

2) Revealing the abnormal hippocampal volume and the regulatory role of the Wnt/β-catenin pathway in the pathogenesis of depression at the morphological and molecular levels.

**Feasibility Analysis**

(1) Chronic Unpredictable Mild Stress (CUMS) is a well-established animal model for studying depression, as it induces symptoms similar to those observed in depressed patients. The project applicants and principal investigators are proficient in stress induction methods and can ensure the proper preparation of CUMS rat models.

(2) The research team consists primarily of young researchers, all of whom have experience in leading teaching and research projects, and can ensure sufficient working time to complete the required tasks.

(3) The applicant's laboratory is equipped with common devices and instruments, sufficient to meet experimental requirements.

(4) The project applicant has been engaged in basic research on stress-related diseases in recent years, is familiar with the cutting-edge theories and related research on stress-related diseases such as post-traumatic stress disorder and depression, and has published some research papers, providing a solid research foundation for this project.

Additionally, this study draws on a large body of literature and combines the applicant's previous research foundation to further explore and deepen the research.

Therefore, this project possesses favourable conditions in terms of research foundation, experimental conditions, and the experimental techniques required by the research team. With the strong support of the school, we are fully capable of completing this project.
